# The Case for Heat Acclimatization of Disaster Responders—An Australian Perspective

**DOI:** 10.3389/fpubh.2017.00098

**Published:** 2017-04-25

**Authors:** Matt B. Brearley, Ian N. Norton, Abigail S. Trewin

**Affiliations:** ^1^Disaster Response and Preparedness, National Critical Care and Trauma Response Centre, Darwin, NT, Australia; ^2^Emergency Risk Management and Humanitarian Response, World Health Organisation, Geneva, Switzerland

**Keywords:** acclimatization, disaster, emergency, heat stress, preparedness, responder, tropics

Responding to sudden onset disasters in South East Asia and the Western Pacific requires teams of medical personnel to work in hot and seasonally humid environments. Recent Australian Medical Assistance Team (AusMAT) deployments post Typhoon Haiyan (Philippines, 2013), Tropical Cyclone Pam (Vanuatu, 2015), and Tropical Cyclone Winston (Fiji, 2016) exposed responders to such conditions. With the exception of Australia’s northern tropics that experience hot weather year round (1), such conditions are in contrast to the temperate climate experienced by the vast majority of Australians. In order to assemble highly skilled, trained, and accredited AusMAT, members may be recruited from all Australian states and territories, creating the potential for disparity between the climate that team members are accustomed to living and working in, and that of the deployment region. This is an important workplace health and safety consideration, as team members are expected to work 12 h shifts over 14 consecutive days (2). Sustained exposure to hot and humid work conditions increases the risk of heat stress, impacting both individual team member health and the delivery of medical services to those in need. While strategies are available to mitigate responder heat stress (3–6), most approaches are resource dependent and may therefore not be feasible in austere disaster settings. By contrast, heat acclimatization, defined as the physiological and perceptual adaptations conferred by frequent elevations of core temperature, is a mitigation strategy that can be developed and maintained in the home environment in readiness for deployment.

A recent report from hot and humid field conditions (mean 34°C, 48% relative humidity) highlighted the value of heat acclimatization in a simulated disaster setting by comparing physiological variables of heat acclimatized (HA) and non-heat acclimatized (NHA) responders. Despite similar physiological values during the initial stages of disaster response, HA responders sustained a significantly higher mean core temperature (38.5°C) than their NHA counterparts (38.1°C) (7). This seemingly counterintuitive outcome is in contrast to laboratory-based fixed workload research that results in less physiological perturbation following completion of a structured heat acclimatization program (8). The key discrepancy between settings is the self-paced workload, allowing HA responders to seemingly adopt a higher output, greater metabolic heat production, and body heat storage than the NHA cohort. Interestingly, both cohorts reported their thermal sensation as “hot” and thermal discomfort as “uncomfortable” despite the core temperature discrepancy, symbolic of both groups regulating their work output according to how hot they felt (9). The apparent dissociation between core temperature and thermal sensation was likely due to the chronic exposure to hot working conditions of the HA responders, habituating them to feeling warm/hot. With similar pre-shift hydration status and overall dehydration, differences between cohorts could not be attributed to hydration. Despite their apparent pacing of effort, 63% of the NHA cohort reported ill to the onsite medical team during the simulation compared to 25% of HA responders. In summary, HA responders demonstrated superior heat tolerance than their NHA counterparts.

Currently, heat acclimatization is not formally recognized alongside accreditation, vaccination, medical clearance, and completion of deployment training as prerequisites for AusMAT deployment. Reports of disaster responders suffering heat stress (10–12) complement the research evidence supporting deployment of HA responders to hot climates. We therefore make the case for consideration of heat acclimatization in the determination of an individual responders suitability for deployment where heat will be encountered.

The nature of sudden onset disasters precludes provision of substantial notice of initial disaster response. Therefore, responders with year round heat acclimatization would be in a physiological state of readiness to work in hot and humid conditions. Such responders ought to be considered as preferred for deployment as part of the initial response team. For those responders yet to achieve heat acclimatization, the process can be commenced immediately in anticipation of deployment as part of the second response team. In this regard, individualized guidelines have been developed to assist medical responders and were utilized for the 2013 Typhoon Haiyan AusMAT response (13). These guidelines are highly relevant to Emergency Medical Teams (14) from temperate climates deploying to tropical regions.

Objective determination of responder heat acclimatization status requires a laboratory heat tolerance test (15) or field-based equivalent that is currently beyond the scope of AusMAT. However, active promotion of regular physical training for heat acclimatization ought to be considered as a prerequisite for members of Emergency Medical Teams with potential for deployment to the tropics. Where chronic heat acclimatization cannot be maintained, use of the pre-deployment guidelines will promote heat acclimatization to mitigate the risk of responder heat stress.

## Author Contributions

MB conceived idea for manuscript. MB, IN, and AT drafted and revised the manuscript.

## Conflict of Interest Statement

The authors declare that this manuscript was developed in the absence of any commercial or financial relationships that could be construed as a potential conflict of interest.
